# Olfactory tracking strategies in a neotropical fruit bat

**DOI:** 10.1242/jeb.231829

**Published:** 2021-02-19

**Authors:** Alyson F. Brokaw, Michael Smotherman

**Affiliations:** 1 Interdisciplinary Program in Ecology and Evolutionary Biology, Texas A&M University, College Station, TX, USA; 2 Department of Biology, Texas A&M University, College Station, TX, USA

**Keywords:** Olfactory tracking, Olfaction, Tracking behavior, Search strategies, Bats

## Abstract

Many studies have characterized olfactory-tracking behaviors in animals, and it has been proposed that search strategies may be generalizable across a wide range of species. Olfaction is important for fruit- and nectar-feeding bats, but it is uncertain whether existing olfactory search models can predict the strategies of flying mammals that emit echolocation pulses through their nose. Quantitative assessments of how well echolocating bats track and localize odor sources are lacking, so we developed a behavioral assay to characterize the olfactory detection and tracking behavior of crawling northern yellow-shouldered bats (*Sturnira parvidens*), a common neotropical frugivore. Trained bats were presented with a choice between control and banana-odor-infused solutions in a series of experiments that confirmed that bats are able to locate a reward based on odor cues alone and examined the effect of odor concentration on olfactory search behaviors. Decision distance (the distance from which bats made their change in direction before directly approaching the target) was distinctly bimodal, with an observed peak that coincided with an inflection point in the odor concentration gradient. We observed two main search patterns that are consistent with both serial sampling and learned route-following strategies. These results support the hypothesis that bats can combine klinotaxis with spatial awareness of experimental conditions to locate odor sources, similar to terrestrial mammals. Contrary to existing models, bats did not display prominent head-scanning behaviors during their final approach, which may be due to constraints of nasal-emitted biosonar for orientation.

## Abstract (precis)

**Summary:** Characterization of the olfactory tracking behavior of northern yellow-shouldered bats revealed that the bats can localize an odor reward using klinotaxis and strategic route following, but display fewer head-scanning movements than other mammals.

## INTRODUCTION

Olfactory search trajectories show striking similarities across diverse taxa, suggesting that many species have converged upon a similar sequence of behaviors to solve the problem of locating an odor source in a dynamic environment (Ache and Young, 2005; Svensson et al., 2014). Examples from many animals have revealed a multi-tiered search strategy to detect and follow odors to their source that relies upon a combination of serial sampling (klinotaxis) and zig-zag ‘casting’ behaviors far from the source that is replaced by more side-to-side head-scanning movements and stereo-olfaction (tropotaxis) when near the odor source (Baker et al., 2018; Catania, 2013; Liu et al., 2020; Louis et al., 2008; Thesen et al., 1993). Bats offer an interesting test of the generality of this behavioral sequence in mammals because of their aerial nature, high speeds and potential morphological and physiological constraints associated with echolocation.

Odor cues play a key role in foraging by frugivorous and nectarivorous bats (Korine and Kalko, 2005; Rieger and Jakob, 1988; Thies et al., 1998; Von Helversen et al., 2000), but the extent to which bats rely upon olfaction to find food is still unknown. Olfaction was shown to be an important cue for detecting the presence of ripe fruit (Leiser-Miller et al., 2020; Thies et al., 1998). Neotropical fruit bats are highly sensitive to fruit odors and can discriminate odor qualities and quantities – the first step in being able to recognize a concentration gradient (Laska, 1990a,b). Many bats use olfaction in combination with echolocation, but appear to rely mainly on sonar cues to locate targets once stimulated by the presence of an attractive odor cue (Korine and Kalko, 2005; Thies et al., 1998). Consequently, the abilities and limitations of bats for tracking an odor source exclusively by olfaction remain to be determined.

The chemical gradient emanating from a single source is predicted to follow a Gaussian distribution, with the precise odor structure dependent upon molecular masses, diffusion coefficients and emission rates of the odor cocktail, as well as wind speed, atmospheric stability and distances from odor source (Elkinton and Cardé, 1984). Time-averaged models of odor concentration predict a steep gradient near the odor source that transitions to a shallower gradient farther away from the source (Elkinton and Cardé, 1984; Elkinton et al., 1984; Louis et al., 2008). At distances farther from the odor source, where the odor gradient is shallow or irregular (with peaks in instantaneous concentration; Murlis et al., 2000), animals use klinotaxis to orient towards a chemical source by sequentially sampling as they move through the environment (Dusenbery, 1992). Closer to the source, where the odor gradient becomes steeper, animals can also exploit the simultaneous comparisons of odor intensity (Catania, 2013; Takasaki et al., 2012) or arrival timing (Gardiner and Atema, 2010) between two or more spatially segregated receptors (tropotaxis). Morphological comparisons of nostril widths in bats suggest that the nasal emission echolocation pulses may impose an important constraint on leaf-nosed bats' abilities to exploit tropotactic mechanisms (Brokaw and Smotherman, 2020), leading us to hypothesize that they may rely more heavily on different behavioral strategies to track odors.

Animals can optimize their olfactory search behaviorally, particularly in response to environmental variations in odor concentrations or plume turbulence. Reduction of speed when navigating turbulent flows is also common, as observed in a range of taxa including crabs (Weissburg and Zimmer-Faust, 1994), lobsters (Moore et al., 1991), dogs (Thesen et al., 1993) and coati (Hirsch, 2010). Animals can adjust their sampling strategies for olfactory cues by increasing rates of sniffing (Khan et al., 2012; Porter et al., 2007) or antennule flicks (Koehl, 2006), or by lateral movements of the head, nose or antenna (Gomez-Marin et al., 2010; Ino and Yoshida, 2009; Khan et al., 2012; Liu et al., 2020; Mathewson and Hodgson, 1972; Porter et al., 2007; Thesen et al., 1993). Sensory cues are also combined with cognitive strategies (i.e. learning and spatial cues) during olfactory tracking. We refer to the resulting behavior as ‘route following’ to reflect that the animals can learn the spatial arrangements of their environment (natural or experimental) and can deduce the most efficient routes for inspecting multiple likely source coordinates. For example, mice can use airborne gradients to locate odor rewards, but find rewards faster when relying instead on previous experience (Gire et al., 2016). Bats are known to use spatial memory while foraging (Fleming et al., 1977; Thiele and Winter, 2005), and so may also be able to combine olfactory cues with spatial information to locate odor sources.

**Fig. 1.**
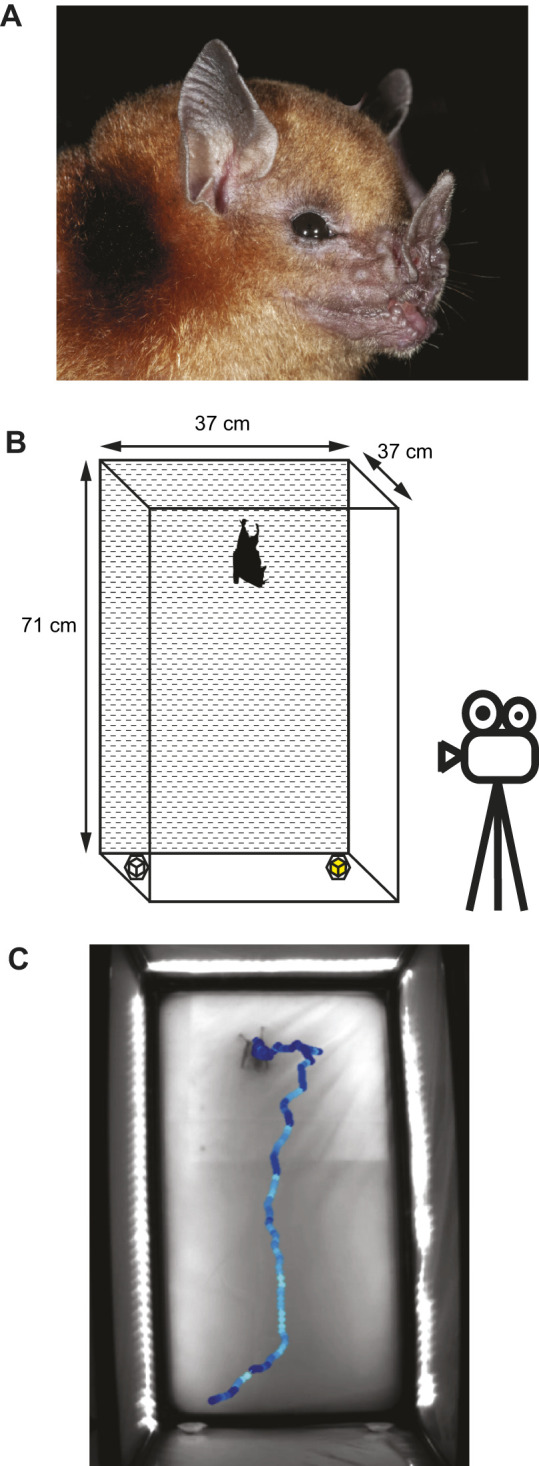
***Sturnira parvidens*, the experimental arena and bat movement analysis.** (A) The northern yellow-shouldered bat. Image by Brock and Sherri Fenton, used with permission. (B) Diagram of the experimental arena used to test bat localization behaviors. The back part of the arena is made of soft mesh for bats to comfortably hang and crawl downward, while the front panel is clear plastic to allow video recording. Shapes at the bottom represent the olfactory stimuli (yellow, S+; white, S−). (C) Example video still and resulting movement track, extracted from EthoVision XT 13. Color gradient represents the bats' instantaneous velocity, with lighter tones representing faster movement. Only trials in which bats crawled along the back of the arena for the entire trajectory were included in movement analysis. Image has been cropped for visualization purposes.

In this study, we quantitatively analyzed the locomotor patterns and behavioral strategies of a phyllostomid bat (Chiroptera: Phyllostomidae) searching for an attractive odor source while crawling downwards. We chose to focus on the northern yellow-shouldered bat, *Sturnira parvidens* (Goldman, 1917) (Fig. 1A), because of its diet, wide distribution and use of olfaction for social communication (Faulkes et al., 2019; González-Quiñonez et al., 2014). The northern yellow-shouldered bat is a small frugivore (13–18 g) common to much of Central America (Hernández-Canchola et al., 2020). This species feeds on a variety of fruits, including banana, wild fig (*Ficus*) and neotropical fruits in the genus *Solanum* (including *S. hazenii*, *S. angulate*, *S. americanum* and *S. torvum*; Castro-Luna and Galindo-González, 2012; Fleming et al., 1977)*.* Field observations suggest that these bats may first use olfactory cues in flight to identify trees bearing ripe fruit, prompting them to land and crawl along branches, where they may rely upon olfaction to find fruit obscured by foliage. Preliminary behavioral experiments confirmed that *Sturnira* readily sought out food in an experimental setting without requiring extensive training, and thus could provide a useful model for measuring bat olfactory tracking capabilities and characterizing their locomotor search strategies. First, we established that naïve crawling bats would successfully localize an attractive odor source in the absence of salient biosonar cues. We then analyzed the locomotor search patterns by quantifying trajectories, speeds and head-scanning behaviors throughout the search to provide a comprehensive characterization of their odor-localization strategies across experimental conditions.

## MATERIALS AND METHODS

### Field conditions

We conducted field experiments from 23 April to 2 May 2019 at Lamanai Outpost Lodge, Orange Walk, Belize (17°45′N; 88°39′W). Bats (*Sturnira parvidens* Goldman 1917) were captured using mist nets from along forest trails and clearings in the Lamanai Archeological Reserve (within 2 km of the Lodge). On the night of capture, we placed individual bats in the experimental arena for 1–2 h with several pieces of banana in plastic hexagonal weigh boats on the floor of the arena. Only individuals that spontaneously sought out and consumed the banana reward by the end of this trial period were retained for behavioral experiments, resulting in *N*=10 male bats. We only used adult male bats in this study to reduce potential confounding factors of sex or age.

### Ethical note

Experiments were carried out under permits from the Belize Forestry Department [permit number FD/WL/1/19 (10)] and were approved by the Texas A&M University Institutional Animal Care and Use Committee (AUP 2017-0139). Care and use of experimental animals complied with relevant local and institutional animal welfare laws, guidelines and policies. Between experiments, bats were housed together in soft mesh cages (60.9×60.9×91.4 cm) in a dark, quiet location and provided water *ad libitum*. During the first 24 h following capture, bats had access to small bowls containing ripe banana at the bottom of the cage. We released all bats at their capture site after a maximum of 5 days.

### Experimental assays

We measured olfactory localization behavior in naïve bats using a two-choice olfactory assay and standard operant procedures. The testing arena was a soft mesh cage (37×37×71 cm) oriented vertically to allow bats to hang and move naturally (Fig. 1B). Pilot behavioral experiments conducted in Belize in 2018 found that bats were more motivated to investigate a possible food reward when allowed to crawl vertically as opposed to crawling horizontally on a surface, as this more closely mimics natural hanging and crawling conditions (such as might be seen in a roost). The front face of the cage was made of clear plastic to allow video recording. Experiments took place between 20:00 h and 06:00 h and were video-recorded with a Basler Ace model ac640-µm digital video camera connected to a laptop running Basler Video Recording Software (Ahrensburg, Schleswig-Holstein, Germany). Videos were recorded at 30 frames s^–1^ and 640×480 pixel resolution. We ran all experiments in complete darkness, except for illumination with infrared light-emitting diode strips attached to the sides of the arena, to remove any confounding visual cues. At the beginning of each trial, bats were placed at the top center of the arena, and stimuli were presented in small plastic bowls (2.5 cm diameter weigh boats), placed at the bottom on opposite sides of the arena. Rewarded stimuli (S+) included real banana pieces or a chemical olfactory cue mixed with sugar water. Chemical olfactory stimuli were prepared using food-grade banana baking emulsion, composed of artificial and natural flavors (LorAnn Professional Kitchen, Lansing, MI, USA). We prepared four concentrations of banana solution using serial dilution, adding 1 ml of banana emulsion (or resulting dilution) to 9 ml of 30% (w/w) sugar solution. All dilutions were prepared from the same batch of banana emulsion and sugar solution. Neotropical bats can discriminate between natural and artificial banana odor (Laska, 1990a), but will still readily consume artificial banana (A.F.B., unpublished observations). We chose to use a baking emulsion as an olfactory cue instead of a pure chemical compound (such as isoamyl acetate) to allow bats to safely consume or taste a reward, in order to maintain motivation during the behavioral trials. During the acclimation and initial training period following capture, we presented bats with banana pieces supplemented with 10% banana–sugar solution to ensure that bats associated the artificial olfactory stimulus with the real banana reward. Unrewarded stimuli (S−) were distilled water or an unflavored piece of sponge cut to mimic the shape and texture of a piece of banana.

In preliminary experiments, we placed a condenser microphone (model CM16, Avisoft Bioacoustics, Berlin, Germany) at the base of the arena to record bats’ echolocation behavior during the olfactory searches. We only detected a bat's broadband echolocation calls when the bat was very near to and directly facing the microphone. However, we noted that the nose-leaf twitched every time the bat emitted a pulse, and based on this it was evident that the bats were continuously emitting pulses whenever they were moving. Because we could not reliably record the pulses throughout the arena as the bats moved, we did not try to quantify their echolocation beyond confirming that they actively echolocated throughout all trials.

The following experiments were designed to evaluate the olfactory search behaviors used by bats locating an odor cue. The first experiment was designed to ensure that naïve bats would reliably seek out a familiar food reward possessing a strong olfactory cue in the test chamber. In the second set of experiments, we controlled for the possible effects of echolocation during olfactory search by testing whether bats could locate the S+ in the absence of salient sonar acoustic cues, by presenting an unscented shape or removing shape cues completely. In the third experiment, we tested the effect of changing odorant concentration on the bat's olfactory localization performance (Table 1). Lastly, we used bat movement trajectories from all experiments to quantitatively describe the behavioral search strategies of crawling bats.

**Table 1.**
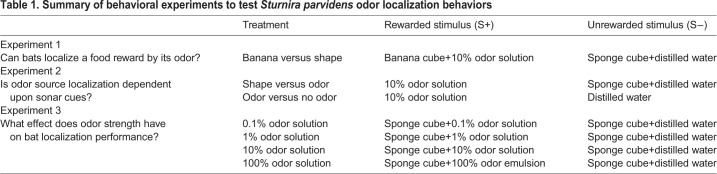
**Summary of behavioral experiments to test *Sturnira parvidens* odor localization behaviors**

### Acclimation and training

On the first night after capture, we introduced naïve bats to the arena and gave them up to 2 h to explore the cage and find the banana food rewards. Bats were gently repositioned by hand at the top of the arena each time a new piece of banana was added to the dish to acclimate them to being handled and reinforce the goal-seeking behavior. Most, but not all, bats quickly learned the task after one night, allowing experimental trials to begin on the second night. Bats that did not seek out food within the arena on the first night were released at their capture site the following night. To reinforce the behavior each night, each experimental session began by presenting the bats with two banana pieces supplemented with 0.5 ml of 10% banana extract solution, which was done to ensure that the bats would associate the extract banana smell with real banana reward even if the bats perceived a difference between extract and real banana smell. We allowed bats to explore the arena until they located and consumed both pieces of banana. We recorded the location where the bat found the first banana. For the subsequent experimental trial, the olfactory stimulus was switched to the opposite side to discourage side bias.

### Experimental animals

On a given night, in-between trials, bats were held individually in soft, cloth bags in a quiet area. Each night, we randomized the order that individuals were tested. The arena was wiped with 95% ethanol and allowed to dry between trials to reduce confounding odor cues. Although experiments are presented and analyzed separately, the trials for all three experiments were randomized within and across nights, to avoid potential confounding effects of learning and maximize sampling across limited individuals and time. We aimed to test each individual 10 times at each treatment. Trials with a banana reward were arranged to be every fourth or fifth trial, to ensure that bats sustained motivation, and so were repeated more than 10 times per individual. The location of S+ was pseudo-randomized for each trial, with its position repeated no more than three consecutive times. We carried out trials under ambient airflow conditions, and temperature and relative humidity were recorded at the start and end of each trial.

### Experiment 1: localization of food reward using odor

During this experiment, bats had the option to choose between a banana reward (S+) and control object (S−). Both choices were placed in plastic weigh boats at the bottom of the arena. We cut ripe bananas into cubes, ∼1 cm^3^. The control object was a cosmetic sponge cut into the same 1 cm^3^ shape as the banana piece. We supplemented the banana reward with 0.1 ml of 10% banana–sugar solution. Both stimuli were prepared and placed in the arena immediately prior to the start of the behavioral trial. We placed an individual bat at the top of the arena to start the trial. Trials lasted until the bat located and consumed the piece of banana, or after a maximum of 5 min had elapsed. If bats did not attempt to feed on the banana after 5 min, then a ‘no-choice’ result was logged.

### Experiment 2: role of acoustic cues during reward localization

The following treatments were designed to isolate olfactory cues from acoustic cues and determine whether or not both sensory modalities (acoustic or odor) were necessary or preferred by the bats during odor localization. In Experiment 2A, we placed 0.5 ml of 10% odor–sugar solution alone (S+) in a plastic weigh boat on one side of the arena, while the other side of the arena held an unscented cosmetic sponge cube cut to resemble a piece of banana placed in 0.5 ml distilled water (S−). This was designed to test which cue type (odor or acoustic) was more important in the bat's search behaviors. In Experiment 2B, we tested how well bats could localize an odor when there was no salient acoustic cue (cosmetic sponge) by placing 0.5 ml of 10% odor–sugar solution (S+) on one side of the arena, while the other side held 0.5 ml distilled water (S−). If bats were successfully able to locate the odor cue, this would provide strong evidence for localization using only odor cues. Trials began when we placed a bat at the top of the arena, and continued until the bat touched, grabbed or licked one of the stimuli. If bats did not select either target after 5 min, then a no-choice result was logged.

### Experiment 3: effect of odor strength on localization success

To evaluate whether or not odor concentration influenced localization performance or search strategies, we challenged the bats with four different concentrations of banana odors. During these experiments, we placed two cosmetic sponge cubes (1 cm^3^) in plastic dishes on opposite sides of the arena. One of the sponges held 0.1 ml of odor–sugar solution (S+), while the other side held a sponge and 0.1 ml distilled water (S−). We tested bats with four different odor concentrations: 100% (only banana extract), 10%, 1% and 0.1%. We determined that bats made a choice when their nose or mouth touched the sponge or weigh boat of one of the stimuli. If bats did not select either target after 5 min, then a no-choice result was logged.

### Behavioral scoring and movement analysis

We recorded every trial for all experiments to analyze and reconstruct the locomotor patterns and pathways used by the searching bats. This information can reveal whether or not the bats consistently used any of the previously defined search strategies seen in other animals (i.e. cast and surge) while tracking odor sources across experimental contexts. We extracted and analyzed bat locomotor patterns and two-dimensional trajectories using Noldus EthoVision XT 13 (Leesberg, VA, USA) (Fig. 1C). The coordinate space was calibrated automatically in EthoVision XT by inputting the real-world height and width of the back of the experimental arena (where bat movement would be measured). The coordinate space was calibrated individually for each video, to account for any movements of either the arena or camera between trials. Bat choices were determined when a bat touched their nose or mouth to one of the stimuli (touching either banana, sponge or weigh boat). Trials were scored a ‘success’ when bats correctly chose the side with the S+. For each trial, we measured or calculated the following: start distance (cm), total distance traveled (cm), mean velocity (cm s^−1^), path straightness, decision distance (cm) and path shape (Table 2). Total distance traveled and mean velocity were automatically calculated in EthoVision XT. We manually measured or classified starting distance, decision distance and path shape from each trial using the integrated tracking view in EthoVision XT. Starting distance and decision distance were calculated in EthoVision XT as the straight-line distance between the bat center point and the odor location at the start of the trial (starting distance), and at the time point at which the bat made its last change of direction before moving towards its target (decision distance). To investigate whether and how bats use head movements during an olfactory localization task, we also analyzed head-scanning behavior for successful trials in Experiment 1 and Experiment 3. A head-scanning event was counted each time the bat rotated its nose at least 45 deg off axis to one side or the other, and these events were only observed to occur consistently when the bat was stationary. Actively crawling bats generally kept their nose-leaf pointed forward in line with the body axis; during locomotion any changes in head orientation were coordinated with concurrent changes in body orientation and therefore not interpreted as head scanning. We extracted the distance from the odor source at which each head-scanning event occurred using EthoVision XT. In addition to counting the total number of head-scanning events, we also recorded the number of head scans that occurred before or after the bat started moving towards the bottom of the arena (starting distance), and before and after the bat made its final decision (decision distance). Head-scanning events at the start or decision distance were counted as occurring ‘after’ this cutoff.

**Table 2.**
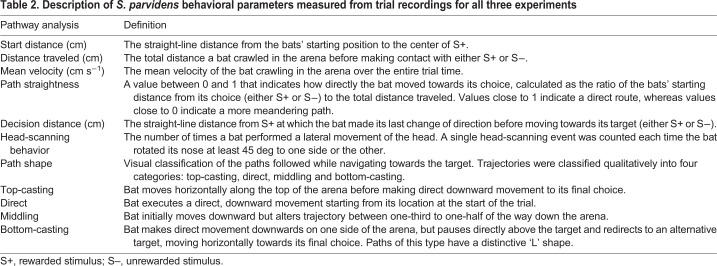
**Description of *S. parvidens* behavioral parameters measured from trial recordings for all three experiments**

**Fig. 2.**
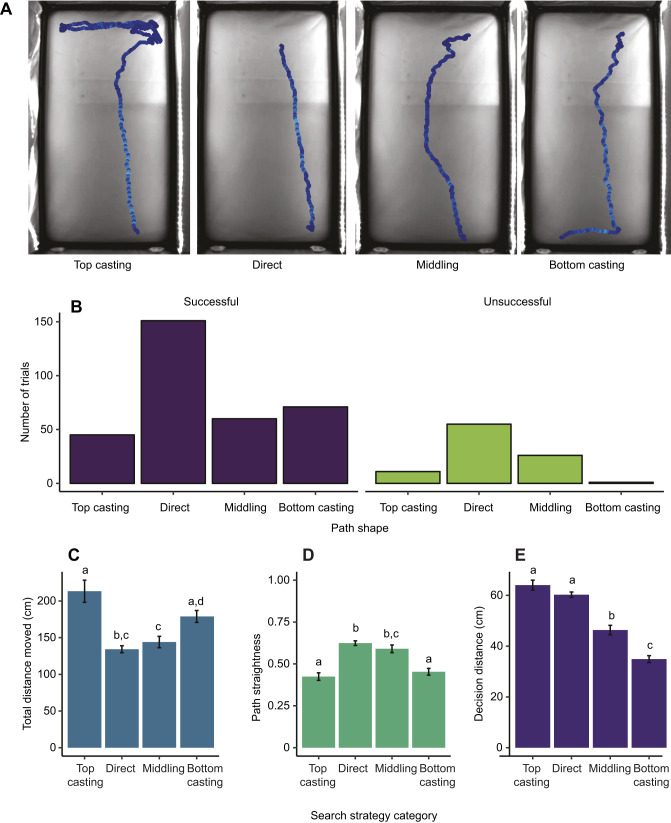
**Path shape characteristics and search strategy categories for *S. parvidens* movement.** (A) Example tracks for each of the four path shapes (Table 2). These tracks were selected from different individuals and different experimental trials. Images have been cropped for visualization purposes. (B) Distribution of the observed number of trials for each path shape for successful (*N*=327) and unsuccessful (*N*=93) trials, pooled across all treatments and individuals. (C) The mean total distance traveled by bats for each search strategy category. (D) The mean path straightness (ratio between starting distance from correct stimuli and total distance moved) across the search strategy categories. Values closer to 1 indicate a straight-line trajectory. (E) The mean distance at which bats made their final decision across all four search strategy categories. Error bars for C-E represent within-individual standard error (‘summarySEwithin’ in package ‘Rmisc’; https://cran.r-project.org/web/packages/Rmisc/index.html). Means with the same letters are not significantly different, according to Tukey's *post hoc* tests (repeated measures ANOVA, α=0.05).

Only trials in which bats remained along the back of the arena until making a choice were used for trajectory analysis and classified into path shapes (Fig. 2). Owing to inaccuracies in tracking introduced by three-dimensional motion, trials in which bats flew or hovered during the trial, or crawled along the side panels of the arena, were excluded from trajectory analysis, although these trials were included in the analyses of bat success rates.

Path shapes were qualitatively classified visually from the detailed tracking view in EthoVision XT 13, and trajectories were defined as one of four categories: top-casting, direct, middling and bottom-casting (Fig. 2A, Table 2)*.* Top-casting was defined as horizontal movement from the bats' starting position at the top of the arena, in which bats crossed the midline of the arena at least once before making a straight path downwards towards one of the stimuli. In direct strategies, bats moved downward without making horizontal shifts in movement. These paths were either straight downward or had a slightly diagonal shape, depending on the bat's exact starting point. Bottom-casting strategies were essentially the inverse of top-casting paths, in which bats made a straight movement downwards towards one of the stimuli, but then moved horizontally across the bottom of the arena (crossing the midline at least once) before making a final choice. Paths in this category produce a distinctive L-shaped pattern. The middling strategy was characterized by general meandering of the path across the arena, in which bats shifted towards the middle of the arena while moving downwards, and then angled diagonally to one of the stimuli between one-third to one-half of the way down the arena (vertical distance).

### Estimating the odor concentration gradient

To estimate the distribution of odors in the arena, we recreated the field setup in the laboratory (College Station, TX, USA) to measure odor concentrations using a handheld photoionization detector (PID) (PhoCheck Tiger, Ion Science, Royston, UK). We placed the same type of plastic weigh boat used in field trials, containing 0.1 ml of 100% banana extract, on one side of the olfactory arena, at the same location at which the odor stimuli were placed during behavioral trials. We divided the back of the olfactory arena into 120 grid spaces, each ∼5.5 cm^2^. Each grid space was measured at 1 s intervals for 5 s, and mean values were calculated for each space. The PID was set to use isoamyl acetate as a standard and was zeroed in clean air using a carbon filter attachment immediately prior to measurements. Because measuring the entire arena would take longer than the maximum time bats were in the arena, we also took measurements of the horizontal and vertical odor distributions at time point zero (immediately following placement of the odor in the arena) and after 5 min, representing the start and end conditions of each trial. Although the laboratory environment is expected to be different from field conditions, the purpose was not to recreate the precise olfactory environment that bats may have been exposed to, which undoubtedly varied slightly between trials, but rather to provide a general estimate for how odors may be distributed within the arena.

### Statistical analysis

The percentages of trials in which the bat correctly chose the odor stimuli (S+) were taken as a measure of performance in all three experiments. Trials in which bats did not select either S+ or S– (no choice) were excluded from analysis. Bat performance between treatments was analyzed using generalized linear mixed models (GLMMs) with a binomial distribution (using glmer in the ‘lme4’ package in R; Bates et al., 2015). Bat ID was included as a random effect to account for repeated testing of individuals. We first tested whether environmental conditions (temperature and humidity) significantly influenced bat performance. We calculated the mean temperature and humidity for each trial [(start value+end value)/2] and analyzed their effect using a GLMM, with temperature and humidity as fixed effects. *Post hoc* tests for significant variables (*P<*0.05) were carried out using Tukey contrasts, adjusted for multiple comparisons (glht in package ‘multcomp’; Hothorn et al., 2008). To test whether the bats were overall able to discriminate better than chance levels within each treatment, we used an intercept-only binomial GLMM predicting bat performance, accounting for repeated measures. In this type of model, the parameter estimate for the intercept can be interpreted to determine whether bats did better than random choice (Maynard et al., 2019). We used one-tailed binomial tests to assess whether individual bats performed better than chance (50%) during the two-choice trials.

To explore how bat strategies varied across trials, we tested whether bat performance could be predicted by certain behavior patterns (such as movement speed, amount of distance traveled or trajectory shape). Search behavioral parameters were log-transformed where appropriate and histograms inspected for outliers before analysis to meet assumptions of normality. We fitted the data to a GLMM with a binomial distribution pooling trials across all experimental treatments (excluding trials in which tracking was unreliable owing to bat flight or the bat leaving the back of the arena). Fixed effects included mean velocity (cm s^−1^), distance traveled (cm), movement time (s), decision distance (cm) and path shape, with bat ID as a random effect. To test the significance of each fixed effect as a predictor of bat performance, we used a model simplification approach (Crawley, 2013). No interactions were included in the models owing to limited sample size. If a significant effect was detected in the model (*P<*0.05), we used a *post hoc* Tukey contrast adjusted for multiple comparisons to examine any differences.

Behavioral strategies are also likely to be context dependent, and individuals can show plasticity in their strategies. To examine how bats may adjust their search behaviors as the difficulty of the task increases, we isolated the successful bat trials from banana and odor solution (concentrations 100% to 0.1%) treatments. We fitted linear mixed models (LMMs) with treatment as an explanatory variable and different trajectory measures (mean velocity, distance traveled, decision distance) as response variables (using restricted maximum likelihood, lme in package ‘nlme’; https://CRAN.R-project.org/package=nlme). Bat ID was included in the model as a random effect to account for repeated testing of individuals.

Finally, we investigated the role of head scanning in bat localization strategies by quantifying head movements during the successful trials when the bats were localizing banana and odor solution treatments. We used a GLMM with a Poisson distribution to test whether there was an effect of treatment or path shape on the frequency of head-scanning events, and used a likelihood ratio test to compare a null model with the fitted model separately for each variable. To test whether bats changed their head-scanning behavior with distance from the odor source, we compared, for each bat, the mean number of head-scanning events that occurred before and after the bat made a decision using a paired Wilcoxon sign-ranked test.

Data obtained from video trajectories and used in analysis are available in Table S1. All analyses were carried out using R (version 3.5.0; https://cran.r-project.org/bin/windows/base/old/3.5.0/) and RStudio (https://rstudio.com/).

## RESULTS

We recorded 648 behavioral assay trials across 10 individual bats and seven experimental treatments. Bats made a choice (correct or incorrect) in 529 trials. Owing to limitations in the field, the number of trials for each treatment for each bat was not equal. The minimum number of trials recorded for a treatment was five and the maximum number of trials for a treatment was 19. All 10 bats were tested across all experimental treatments except for three individuals, which were not exposed to the odor-only treatment.

Temperature was fairly consistent across all trials (27.9±0.04°C) (values provided as means±s.e.m.) and did not have a significant effect on bat performance (all trials pooled, binomial GLMM, *z=*1.414, *P=*0.158). Relative humidity varied slightly more across trials (70.4±0.11%) and did have a significant effect on bat performance (all trials pooled, binomial GLMM, *z=*−2.032, *P=*0.042). To account for this variation, mean relative humidity was included as a random effect in the generalized linear mixed models. There was also no effect of trial order on performance; that is, bats were not more successful at localizing odors in later trials than in trials early in the experiment (all trials pooled, binomial GLMM, *z*=−1.491, *P=*0.136).

### Experiment 1: localization of food reward using odor

In this experiment, we established whether bats could consistently and successfully locate a rewarded odor. Bats were reliably able to locate the location of a rewarded odor, with eight out of 10 individuals performing above chance in a two-choice assay (one-tailed binomial test, *P<*0.05, *N*=10 bats, 120 trials, 8–18 trials per bat). For the two bats that did not perform better than chance, they only made a choice during three (Bat 7) and six (Bat 8) out of 10 trials, suggesting low motivation and not lack of tracking ability. On average, bats successfully located the odor reward 90.7±6.99% of the time (excluding trials in which bats did not make a choice), exhibiting non-random preference for the odor-rewards side (intercept-only binomial GLMM, *P<*0.01).

### Experiment 2: role of acoustic cues during reward localization

**Fig. 3.**
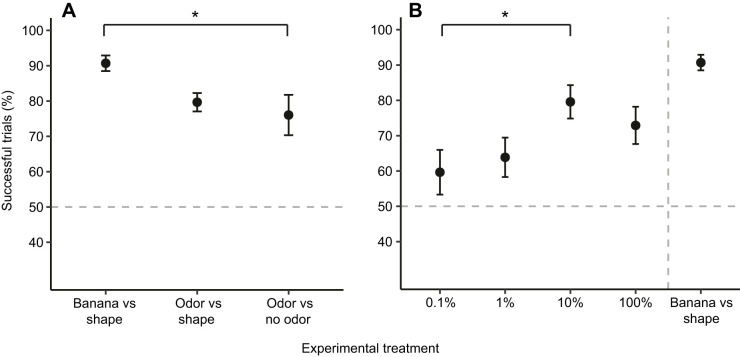
***S. parvidens* success rates in localizing the rewarded** **stimulus across treatments for Experiments 1–3.** (A) Comparison of the mean success rate [percentage of trials in which bats correctly chose the rewarded stimulus (S+)] across treatments for Experiments 1 and 2. Dots and bars indicate means±s.e.m. Bats performed better than expected by chance in all three treatments (intercept-only GLMM, *P*<0.05). A significant difference in performance between banana versus shape and odor versus no odor treatments was observed (binomial GLMM with repeated measures, *P<0.05). (B) Comparison of the mean success rate across treatments for Experiment 3 (percentage banana concentration). Dots and bars indicate means±s.e.m. Bats performed better than expected by chance when localizing 1%, 10% and 100% banana concentrations (intercept-only GLMM, *P*<0.05). A significant difference in performance between the 1% and 10% treatments was observed (binomial GLMM with repeated measures, *P<0.05). The results from the banana treatment were not included in the analyses of this experiment, but are included in this graph as a reference. Dashed horizontal lines in A and B indicate chance levels of success (50%).

In the first part of this experiment (Experiment 2A), we tested whether bats would localize an attractive odor cue without the appropriately matching echolocation cue. On average, bats performed better than chance at locating the odor-rewarded side, even when there was not an accompanying shape cue (intercept-only binomial GLMM, *P<*0.01) and successfully chose the odor cue in most of the trials (79.7±8.24%, *N*=10 bats, 72 trials, 4–9 trials per bat). In the second part of the experiment (Experiment 2B), we tested whether bats could successfully locate an odor cue when no salient echolocation cues were present, by removing shape cues (i.e. banana piece or cosmetic sponge). Again, bats performed better than chance at locating the odor-rewarded side in both treatments (intercept-only binomial GLMM, *P<*0.01 for both treatments). The mean success rate for bats localizing an odor without a distinctive echolocation target was lowest compared with the mean success rates for other experimental treatments (76±5.72%, *N*=7 bats, 64 trials, 3–11 trials per bat). Comparing bat performance across treatments from Experiments 1 and 2, experimental treatment had an effect on localization success across all 10 bats (binomial GLMM, *F=*3.5308, d.f.=2). Bats were more successful at locating the banana reward compared with their performances in trials in which there were no distinctive echolocation cues available to guide them (*z*=−2.652, *P*=0.0217) (Fig. 3A). Neither start latency (time at top of the arena before moving downward) or decision distance had an effect on bat performance.

### Experiment 3: effect of odor strength on localization success

We also tested how decreasing odor concentrations would affect bat olfactory localization performance. Overall, bats performed better than chance when locating 100%, 10% and 1% odor concentrations (intercept-only binomial GLMM, *P<*0.01 for all three treatments). Percentage success decreased with a decrease in concentration, and bats had the highest mean success rate when localizing the 10% odor solution (79.57±4.73%). Bats were least successful when searching for the 0.1% odor solution, particularly when compared with the 10% odor solution (*z*=2.838, *P=*0.0233, Fig. 3B). Although four out of 10 bats performed better than predicted by chance at locating the 10% concentrations (binomial one-tailed test, *P<*0.05), we did not have sufficient power to make conclusions on individual performance owing to limited trial sample sizes for most individuals (3–11 trials per bat, per treatment after no-choice trials were removed).

### Behavior and movement analysis

Across all experiments, we analyzed bat movements to quantify and categorize the potential odor localization strategies bats are using to localize an odor source. Only trials in which bats crawled along the back of the arena to reach their choice (S+ or S−) were included in this analysis (*N*=420 trials, e.g. Movie 1), consisting of 79% of all recorded trials in which bats made a choice (420/529 trials). Of the analyzed trajectories, 53.1% of trajectories were from trials in which the odor was presented on the left side of the arena (223/420 trials) and 46.9% were trials in which the odor was presented on the right (197/420 trials).

**Fig. 4.**
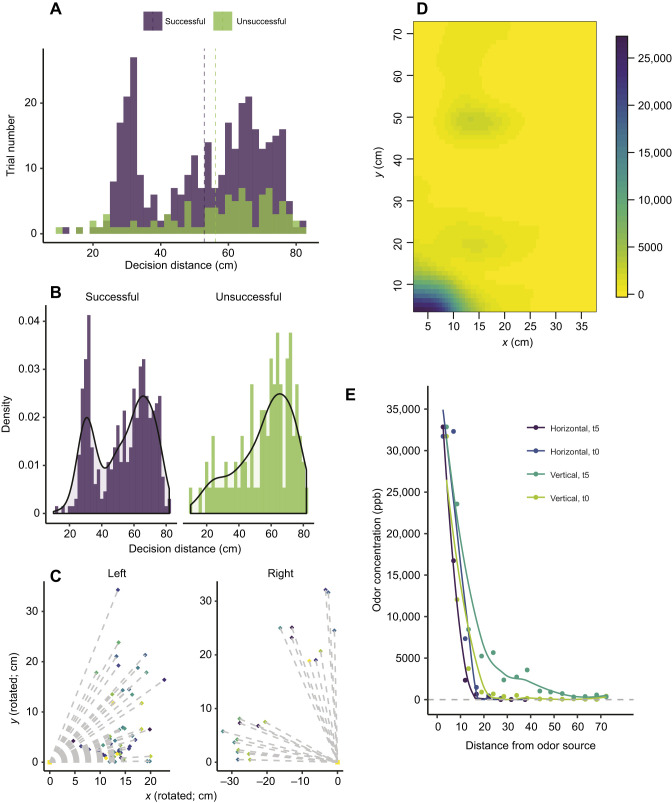
**Distributions of *S. parvidens* decision distances in trials and changes in odor concentrations of the olfactory stimulus with distance from the source.** (A) Distribution of decision distances across trials, shown for both successful and unsuccessful trials (trials pooled across all experimental treatments and individuals, *N*=420 trials). Dashed vertical lines represent the mean decision distance for each category (successful, unsuccessful). (B) Distribution of decision distances, normalized by density for successful trials (blue, *N*=327 trials) and unsuccessful trials (pink, *N*=93 trials). (C) Graphical plot of the pooled trials with decision distances (diamonds) within 25–35 cm of the odor stimulus (squares). Different colors represent trials of different individuals. The left side represents trials in which the odor stimulus was on the left side of the arena (*N*=60 trials), and the right side represents trials in which the odor stimulus was on the right side (*N*=21 trials). Coordinates were obtained from EthoVision XT 13, then rotated and transformed to standardize the location of the odor stimulus (based on a Cartesian coordinate system). (D) Heat map with a Gaussian smoothing function (smooth.2d, theta=4 in package ‘fields’; https://github.com/NCAR/Fields), representing the measured concentrations [in parts per billion (ppb)] measured within the arena when 0.1 ml 100% banana extract was placed on the left side. (E) Plots showing the decay of odor concentration of 100% banana extract (in ppb) with distance from the odor source along the horizontal (purple) and vertical (green) axes of the behavioral arena immediately after odor placement (t0) and 5 min after odor placement (t5).

We log-transformed mean velocity and total distance traveled to meet assumptions of normality. Inspection of the distribution for decision distance revealed a bimodal distribution (Fig. 4A). When separated between successful and unsuccessful bat trials, there was a peak in the number of successful trials in which bats made their decision between 25 cm and 35 cm from the stimulus (Fig. 4B,C). This bimodality was not seen when looking only at unsuccessful trials (Fig. 4B). Neither distance traveled nor mean velocity had a significant effect on bat performance, but there was a significant relationship between bat performance and trajectory shape (GLMM, *F=*4.067, d.f.=3).

Looking only at trials in which bats successfully located the banana odors, there was a significant difference in the log-distance traveled during the trial, log-mean velocity and decision distance across treatments (excluding treatments from Experiment 2, *N*=327 trials) (LMM, *P<*0.05). Bats traveled a shorter distance when localizing the 10% odor concentration compared with the 1% odor concentration (*z*=−3.411, *P=*0.005) and banana (*z*=2.86, *P=*0.034) treatments. Bat trajectories were also more direct (as measured by straightness) when localizing the 10% odor concentration compared with the 1% odor concentration (*z*=3.083, *P=*0.0176) and banana (*z*=−3.016, *P=*0.0214) treatments. Bats also moved fastest when navigating towards the 10% odor concentration, particularly when compared with the banana treatment (*z*=−2.753, *P=*0.046). Decision distance was variable across treatments, with bats making their final decision closer to the banana stimuli compared with the 100% odor concentration (*z*=−2.988, *P=*0.0214).

All four locomotor patterns were observed in successful trials across treatments, but bottom casting was significantly more frequent in successful bats than in unsuccessful bats (*z*=2.688, *P<*0.01) (Fig. 2B). All individuals used each of the four search strategies at least once. To validate our qualitative categorization of search strategy, we compared the total distance traveled, path straightness and decision distance using a repeated-measures ANOVA (with bat ID as a random factor). Straightness was significantly different between path shapes (*F*=21.09, *P<*0.001, Fig. 2D). Both direct and middling strategies were significantly straighter than either casting strategy (Tukey's pairwise comparison, *P<*0.001) but were not significantly different from each other (*t=*−1.183, *P=*0.634). Similarly, straightness of the top-casting and bottom-casting strategies did not differ significantly from each other (*t=*1.143, *P=*0.660). However, bats did travel significantly further (total distance) when using the top-casting strategy compared with all other strategies (*P<*0.05 in pairwise comparisons, Fig. 2C). The decision distance was not significantly different between top-casting and direct strategies (*t=*−2.041, *P=*0.171), but both were significantly farther away from the correct stimuli compared with the other two strategies (pairwise comparison, *P<*0.001, Fig. 2E). Based on these differences, we conclude that the strategies are qualitatively and quantitatively different from each other. The top-casting strategy is characterized by the farthest traveled distance, the furthest decision distance and least straight trajectory compared with the other three strategies. Although similar to top casting in straightness and total traveled distance, bottom casting had the closest decision distance of all four strategies. By contrast, the direct strategy was the straightest path observed in these trials, and bats made their decision at similar distances compared with top casting. Although similar to the direct strategy in straightness and total distance traveled, the decision distance for the middling strategy was closer to the correct stimuli, but not as close as in bottom-casting trajectories.

**Fig. 5.**
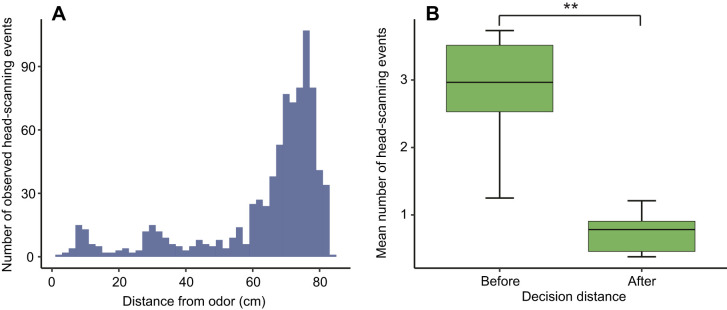
***S. parvidens* head-scanning behaviors in Experiments 1 and 3.** (A) Distribution of the numbers of individual head-scanning events (*N*=849 events) relative to distance from the odor source, pooled across all successful trials and individuals for Experiment 1 and Experiment 3 (10 bats, 247 trials). (B) Mean number of head-scanning events (mean of all trials for each individual, *N*=10) observed before and after bats made their final change of direction in successful trials in Experiment 1 and Experiment 3. A significant difference in head-scanning behaviors before and after reaching the decision distance was observed (paired Wilcoxon sign-rank test, **P<0.01). Top and bottom horizontal bars of the box represent the 1st and 3rd quartiles, middle horizontal bar represents the median, and whiskers show minimum and maximum values.

To analyze head-scanning behavior, we pooled successful trials from which we were able to obtain high-quality reconstructions of the bats’ trajectories from Experiment 1 (banana) and Experiment 3 (percentage odor concentrations), resulting in a total of 247 trials across 10 individuals (15–40 trials per individual). We observed 849 total head-scanning events across all trials. Most head-scanning behavior occurred at distances 60–80 cm from the odor source, i.e. when the bats were at the top of the arena (Fig. 5A). Bats performed significantly more head scans before starting their downward trajectory towards the odor source (Wilcoxon sign-rank test, *V=*53, *P=*0.005), and before making their final direction decision (Fig. 5B, Wilcoxon sign-rank test, *V=*55, *P=*0.001). Neither concentration nor path shape had an effect on the total number of observed head-scanning events (GLMM likelihood ratio test, *P>*0.05).

### Estimating the odor concentration gradient

Odors in the arena were not evenly distributed, but the odor structure in the arena was consistent with a Gaussian distribution, with the highest concentrations recorded immediately above and next to the odor stimulus. The odor concentrations declined rapidly with distance from the odor in both horizontal and vertical directions (Fig. 4D). After 5 min (the maximum trial time), odor concentrations along the vertical axis stayed either constant or increased, staying higher along the middle of the arena compared with the horizontal odor distribution. Along the horizontal axis, the odor concentration gradient dropped close to 0 (or below detectable levels using the PID) ∼30 cm from the odor source, but did not drop to 0 until 35–55 cm in the vertical direction (Fig. 4E).

## DISCUSSION

Our results suggest that bats use klinotactic olfactory tracking strategies similar to other terrestrial mammals, including humans (Jinn, 2019), mice (Gire et al., 2016; Liu et al., 2020) and rats (Bhattacharyya and Bhalla, 2015). Although previous work demonstrated that bats are able to detect and discriminate concentration gradients to localize odor rewards (Laska, 1990a,b), this is the first study to specifically quantify the locomotor patterns and olfactory search strategies of bats. Similar to previous research demonstrating the importance of olfactory cues in other echolocating and non-echolocating bat species (Hodgkison et al., 2007; Korine and Kalko, 2005; Parolin et al., 2015; Tang et al., 2007; Thies et al., 1998; Von Helversen et al., 2000), northern yellow-shouldered bats were able to localize an odor reward using olfaction under experimental conditions that controlled for echolocation cues. By recording the bats’ movements in an open-field-type behavioral setup (as opposed to a Y-maze or other choice paradigm), we were able to exploit this behavior and quantitatively describe the search routes bats followed while localizing an odor reward. We showed that bats were able to find odor sources even when the measured concentration of odors in the air was very low, consistent with previous studies on bat olfactory sensitivity (Laska, 1990b), which reported detection thresholds in the range of ∼3–15 parts per billion.

Olfactory localization strategies are often multi-modal, with animals integrating olfactory cues with visual, mechanosensory and acoustic inputs (Cardé and Willis, 2008; Gomez-Marin et al., 2010; Vickers, 2000). Although bats, including neotropical leaf-nosed bats, use vision as part of their orientation and foraging strategy (Gutierrez et al., 2014), it is unlikely that visual cues provide much detailed information. Most bat-dispersed fruits in the neotropics do not change color with ripening, opposite to the pattern observed in many bird- and primate-dispersed plant species (Kalko et al., 1996; Lomáscolo et al., 2010). Visual cues and acoustic cues are also less reliable against cluttered backgrounds (such as a fruit cluster on a leafy branch), and it has been shown that removal of visual cues does not significantly impact bat foraging success (Korine and Kalko, 2005; Thies et al., 1998).

Like other neotropical leaf-nosed frugivores, *Sturnira* produces low-intensity, high-frequency echolocation calls, with peak frequencies ranging from 65 kHz to 92 kHz (Jennings et al., 2004; Yoh et al., 2020), emitted via the nose. Fruit- and nectar-feeding bats within the family Phyllostomidae (including *Sturnira*) are thought to primarily use echolocation for general orientation, as well as the final approach and selection of food items (Gonzalez-Terrazas et al., 2016; Kalko and Condon, 1998; Leiser-Miller et al., 2020; Thies et al., 1998). We controlled for potential effects of echo-acoustic information during odor localization in Experiment 2. Bats performed better than expected by chance, even when the echolocation cue was paired with the non-rewarded, no odor control (Experiment 2A) and there was no obvious echolocation cue (Experiment 2B) (Fig. 3A). Based on these results, we conclude that acoustic cues did not significantly contribute to the bats’ ability to discriminate the odorized targets, and that the primary sensory cue bats were using in these assays was olfaction. This is further supported by observations that, even when bats chose the wrong side (S–), they did not attempt to consume the control sponge, which would be predicted to have the same acoustic signature at the S+ sponge, whereas they often bit and tasted the banana-scented sponge.

We observed a peak in decision distance at 25–35 cm from the odor source for successful attempts across all concentrations. This distance coincided with an inflection in the steepness of the odor gradient, which provided optimal conditions for bats to detect spatial differences and orient towards the higher concentrations. At distances at which the odor is detectable but the concentration gradient is still shallow, large movements or changes in direction (casting) are more efficient (Catania, 2013). Once the gradient becomes steeper near the source, short movements, head scanning and bilateral inputs may be sufficient to find an odor source (see, for example, fig. 7 in Catania, 2013; Jinn, 2019). That this distance is also about the same as the observed olfactory decision distance of mice following an odor plume (Liu et al., 2020) suggests that this may be a common pattern across mammals.

Our trajectory analysis identified four distinctive search locomotor patterns routinely displayed by all bats within the experimental chamber (Fig. 2A). Because bats are also expected to perform this task in flight at high velocities, we anticipated the possibility of exaggerated or unusual locomotor patterns relative to terrestrial mammals such as dogs or rodents. Contrary to expectations, none of the recorded tracks exhibited the forward zig-zag pattern that characterizes the olfactory tracking trajectories displayed by walking mammals or flying insects (Svensson et al., 2014; Vickers, 2000). The least common pattern, top casting, had broad lateral movements back and forth across the top of the arena that could be characterized as zig-zagging, but these zig-zag motions rarely resulted in net forward motion. This type of movement at the edges of a concentration gradient are consistent with the model posed by Catania (2013), with large movements and serial sampling helping to provide directional information in shallow gradients. The most commonly observed successful locomotor pattern was the direct strategy, representing a relatively direct search pattern with no major changes in orientation during the track (Fig. 3A). Assuming bats are receiving motivational cues from the top of the arena, then this strategy could be compared with the ‘aim-and-shoot’ strategy used by some flying insects to locate odor sources (Cardé and Willis, 2008), which does not always result in a successful search, similar to what we observed in our experiments. This downward movement can be paired with serial sampling as observed in other taxa (Catania, 2013; Liu et al., 2020), allowing the animal to more accurately reassess the direction of the odor gradient when they get nearer the odor source (Jinn, 2019; Thesen et al., 1993). This behavioral strategy is also consistent with the middling locomotor pattern we observed, wherein the bats moved down the center of the chamber until they had sufficient directional information within the odor gradient to select the correct direction.

Bats may be able to pair movement with increased active sampling, such as sniffing (Baker et al., 2018; Khan et al., 2012; Vergassola et al., 2007) and simultaneous head scanning (Gomez-Marin et al., 2010; Khan et al., 2012). Sniffing and head scanning improve the efficiency of klinotactic olfactory localization by allowing an organism to maintain its body orientation within an odor plume, while permitting a longer period to sense and integrate the chemical signal (Dusenbery, 1992). Liu et al. (2020) proposed that, at distances far from the source, serial sampling (sniffing) is performed with whole-body movements, which may be replaced by increased head scanning as mice approach the odor source. By contrast, the bats in our study performed most of their head-scanning movements at the top of the arena before moving towards the odor source (Fig. 5A), and these behaviors were only observed when bats were stationary. As these bats use echolocation for orientation (Hernández-Canchola et al., 2020), and bats are known to use head movements to keep biosonar beam projections fixated on obstacles and targets (Surlykke et al., 2009), we were not able to separate head movements associated with sniffing from those associated with biosonar emissions. It remains possible that bats process olfactory inputs during passive breathing and echolocating (Eiting et al., 2014; Wachowiak, 2011), but the predominance of biosonar for navigation may pre-empt the use of head scanning purely for olfactory search. Although more research in this area is needed, this observation represents a key departure from the current synthesis of olfactory search models proposed for mammals (Baker et al., 2018; Catania, 2013; Liu et al., 2020). This, in combination with having narrow nostrils for emitting pulses through the nose, suggests that bats may be constrained in their ability to use stereo-olfaction and head scanning during the final approach phase of olfactory searches.

Trial-and-error or route-following strategies could help bats overcome the trade-offs between echolocation and serial sampling. Of the four locomotor patterns observed, bottom casting appeared to be consistent with what has been termed route following in other animals. This strategy consisted of rapidly approaching one of the targets and coming within several centimeters of S– before sharply changing direction towards the S+, which suggests that the bats were following a route with a limited number of known options. Under natural foraging conditions, animals supplement sensory information, such as olfactory cues, with long-range navigation and cognitive strategies. Studies in rats have demonstrated that, under certain circumstances (e.g. small number of targets and known locations), strategies such as route following are faster and more robust than gradient following or casting (Bhattacharyya and Bhalla, 2015; Gire et al., 2016), particularly as familiarity with the task increases (Gire et al., 2016). In our assay, there were only two possible locations for the odor reward, which with experience shifts the olfactory task from ‘where’ to ‘which’ (Bhattacharyya and Bhalla, 2015). Bats, particularly nectar-feeding bats, have been shown to have extraordinary spatial working memories (Henry and Stoner, 2011; Toelch et al., 2008; Winter and Stich, 2005). Short-tailed fruit bats (*Carollia*) rely more strongly on spatial memory than sensory cues when foraging in the wild (Fleming et al., 1977), and spatial memory may even overshadow the use of sensory cues such as odors (Carter et al., 2010). Bat flight is also metabolically expensive, so relying on spatial memory and returning to quality foraging locations may be more efficient for foraging fruit bats than following odor plumes, provided they are exploring a known space.

Flying bats are exposed to highly variable olfactory environments when foraging under natural conditions, but they also use olfaction while crawling in roosts or when perched in trees, where their movements are slow and the local olfactory landscape is more stable. Our results suggest that, when bats are restricted to crawling, they display olfactory tracking strategies similar to those of other terrestrial mammals, with only minor constraints arising from echolocation. Future work quantifying how bats navigate towards an odor source while flying would provide more insight into how bats use odors in their natural environment, as well as how use of olfactory sensory cues integrates with other navigational strategies such as echolocation and spatial memory.

## Supplementary Material

Supplementary information

## References

[JEB231829C1] Ache, B. W. and Young, J. M. (2005). Olfaction: diverse species, conserved principles. *Neuron* 48, 417-430. 10.1016/j.neuron.2005.10.022 16269360

[JEB231829C2] Baker, K. L., Dickinson, M., Findley, T. M., Gire, D. H., Louis, M., Suver, M. P., Verhagen, J. V., Nagel, K. I. and Smear, M. C. (2018). Algorithms for olfactory search across species. *J. Neurosci.* 38, 9383-9389. 10.1523/JNEUROSCI.1668-18.2018 30381430 PMC6209839

[JEB231829C3] Bates, D., Maechler, M., Bolker, B. and Walker, S. (2015). Fitting linear mixed-effects models using lme4. *J. Stat. Softw.* 67, 1-48. 10.18637/jss.v067.i01

[JEB231829C4] Bhattacharyya, U. and Bhalla, U. S. (2015). Robust and rapid air-borne odor tracking without casting. *eNeuro* 2, ENEURO.0102-15.2015 10.1523/ENEURO.0102-15.2015 PMC4674010 26665165

[JEB231829C5] Brokaw, A. F. and Smotherman, M. (2020). Role of ecology in shaping external nasal morphology in bats and implications for olfactory tracking. *PLoS ONE* 15, e0226689 10.1371/journal.pone.0226689 31914127 PMC6948747

[JEB231829C6] Cardé, R. T. and Willis, M. A. (2008). Navigational strategies used by insects to find distant, wind-borne sources of odor. *J. Chem. Ecol.* 34, 854-866. 10.1007/s10886-008-9484-5 18581182

[JEB231829C7] Carter, G. G., Ratcliffe, J. M. and Galef, B. G. (2010). Flower bats (*Glossophaga soricina*) and fruit bats (*Carollia perspicillata*) rely on spatial cues over shapes and scents when relocating food. *PLoS ONE* 5, 1-6. 10.1371/journal.pone.0010808 PMC2876041 20520841

[JEB231829C8] Castro-Luna, A. A. and Galindo-González, J. (2012). Enriching agroecosystems with fruit-producing tree species favors the abundance and richness of frugivorous and nectarivorous bats in Veracruz, Mexico. *Mamm. Biol.* 77, 32-40. 10.1016/j.mambio.2011.06.009

[JEB231829C9] Catania, K. C. (2013). Stereo and serial sniffing guide navigation to an odour source in a mammal. *Nat. Commun.* 4, 1441 10.1038/ncomms2444 23385586

[JEB231829C10] Crawley, M. J. (2013). The R Book. In *The R Book*, pp. 388-448. Chichester: Wiley.

[JEB231829C11] Dusenbery, D. B. (1992). *Sensory Ecology: How Organisms Acquire and Respond to Information*. New York: W.H. Freeman.

[JEB231829C12] Eiting, T. P., Perot, J. B. and Dumont, E. R. (2014). How much does nasal cavity morphology matter? Patterns and rates of olfactory airflow in phyllostomid bats. *Proc. R. Soc. B* 282, 20142161 10.1098/rspb.2014.2161 PMC4298208 25520358

[JEB231829C13] Elkinton, J. S. and Cardé, R. T. (1984). Odor dispersion. In *Chemical Ecology of Insects* (ed. W. J. Bell and R. T. Cardé), pp. 73-91. Boston, MA: Springer US.

[JEB231829C14] Elkinton, J. S., Cardé, R. T. and Mason, C. J. (1984). Evaluation of time-average dispersion models for estimating pheromone concentration in a deciduous forest. *J. Chem. Ecol.* 10, 1081-1108. 10.1007/BF00987515 24318851

[JEB231829C15] Faulkes, C. G., Elmore, J. S., Baines, D. A., Fenton, B., Simmons, N. B. and Clare, E. L. (2019). Chemical characterisation of potential pheromones from the shoulder gland of the Northern yellow-shouldered-bat, *Sturnira parvidens* (Phyllostomidae: Stenodermatinae). *PeerJ* 2019, e7734 10.7717/peerj.7734 PMC6754726 31579609

[JEB231829C16] Fleming, T. H., Heithaus, E. R. and Sawyer, W. B. (1977). An experimental analysis of the food location behavior of frugivorous bats. *Ecology* 58, 619-627. 10.2307/1939011

[JEB231829C17] Gardiner, J. M. and Atema, J. (2010). The function of bilateral odor arrival time differences in olfactory orientation of sharks. *Curr. Biol.* 20, 1187-1191. 10.1016/j.cub.2010.04.053 20541411

[JEB231829C18] Gire, D. H., Kapoor, V., Arrighi-Allisan, A., Seminara, A. and Murthy, V. N. (2016). Mice develop efficient strategies for foraging and navigation using complex natural stimuli. *Curr. Biol.* 26, 1261-1273. 10.1016/j.cub.2016.03.040 27112299 PMC4951102

[JEB231829C19] Goldman, E. A. (1917). New mammals from North and Middle America. *Proc. Biol. Soc. Washingt.* 30, 107-116.

[JEB231829C20] Gomez-Marin, A., Duistermars, B. J., Frye, M. A. and Louis, M. (2010). Mechanisms of odor-tracking: multiple sensors for enhanced perception and behavior. *Front. Cell. Neurosci.* 4, 1-15. 10.3389/fncel.2010.00006 20407585 PMC2854573

[JEB231829C21] González-Quiñonez, N., Fermin, G. and Muñoz-Romo, M. (2014). Diversity of bacteria in the sexually selected epaulettes of the little yellow-shouldered bat *Sturnira lilium* (Chiroptera: Phyllostomidae). *Interciencia* 39, 882-889.

[JEB231829C22] Gonzalez-Terrazas, T. P., Martel, C., Milet-Pinheiro, P., Ayasse, M., Kalko, E. K. V. V. and Tschapka, M. (2016). Finding flowers in the dark: Nectar-feeding bats integrate olfaction and echolocation while foraging for nectar. *R. Soc. Open Sci.* 3, 160199 10.1098/rsos.160199 27853595 PMC5108945

[JEB231829C23] Gutierrez, E. de A., Pessoa, V. F., Aguiar, L. M. S. and Pessoa, D. M. A. (2014). Effect of light intensity on food detection in captive great fruit-eating bats, *Artibeus lituratus* (Chiroptera: Phyllostomidae). *Behav. Processes* 109, 64-69. 10.1016/j.beproc.2014.08.003 25153795

[JEB231829C24] Henry, M. and Stoner, K. E. (2011). Relationship between spatial working memory performance and diet specialization in two sympatric nectar bats. *PLoS ONE* 6, e23773 10.1371/journal.pone.0023773 21931612 PMC3170290

[JEB231829C25] Hernández-Canchola, G., León-Paniagua, L. and Org, M. (2020). *Sturnira parvidens* (Chiroptera: Phyllostomidae). *Mamm. Species* 52, 57-70. 10.1093/mspecies/seaa005 28647618

[JEB231829C26] Hirsch, B. T. (2010). Tradeoff between travel speed and olfactory food detection in ring-tailed coatis (*Nasua nasua*). *Ethology* 116, 671-679. 10.1111/j.1439-0310.2010.01783.x

[JEB231829C27] Hodgkison, R., Ayasse, M., Kalko, E. K. V., Häberlein, C., Schulz, S., Mustapha, W. A. W., Zubaid, A. and Kunz, T. H. (2007). Chemical ecology of fruit bat foraging behavior in relation to the fruit odors of two species of paleotropical bat-dispersed figs (*Ficus hispida* and *Ficus scortechinii*). *J. Chem. Ecol.* 33, 2097-2110. 10.1007/s10886-007-9367-1 17929094

[JEB231829C28] Hothorn, T., Bretz, F. and Westfall, P. (2008). Simultaneous inference in general parametric models. *Biometrical J.* 50, 346-363. 10.1002/bimj.200810425 18481363

[JEB231829C29] Ino, Y. and Yoshida, K. (2009). Parallel use of two behavioral mechanisms for chemotaxis in *Caenorhabditis elegans*. *J. Neurosci.* 29, 5370-5380. 10.1523/JNEUROSCI.3633-08.2009 19403805 PMC6665864

[JEB231829C30] Jennings, N. V., Parson, S., Barlow, K. E. and Gannon, M. R. (2004). Echolocation calls and wing morphology of bats from the West Indies. *Acta Chiropt.* 6, 75-90. 10.3161/001.006.0106

[JEB231829C31] Jinn, J. (2019). Orientation and sampling strategies in mammalian olfactory navigation. *PhD thesis*, University of California, Berkley, CA, USA.

[JEB231829C32] Kalko, E. K. V. and Condon, M. A. (1998). Echolocation, olfaction and fruit display: how bats find fruit of flagellichorus cucurbits. *Funct. Ecol.* 12, 364-372. 10.1046/j.1365-2435.1998.00198.x

[JEB231829C33] Kalko, E. K. V., Herre, E. A. and Handley, C. O. (1996). Relation of fig fruit characteristics to fruit-eating bats in the New and Old World tropics. *J. Biogeogr.* 23, 565-576. 10.1111/j.1365-2699.1996.tb00018.x

[JEB231829C34] Khan, A. G., Sarangi, M. and Bhalla, U. S. (2012). Rats track odour trails accurately using a multi-layered strategy with near-optimal sampling. *Nat. Commun.* 3, 703 10.1038/ncomms1712 22426224

[JEB231829C35] Koehl, M. A. R. (2006). The fluid mechanics of arthropod sniffing in turbulent odor plumes. *Chem. Senses* 31, 93-105. 10.1093/chemse/bjj009 16339271

[JEB231829C36] Korine, C. and Kalko, E. K. V. (2005). Fruit detection and discrimination by small fruit-eating bats (Phyllostomidae): echolocation call design and olfaction. *Behav. Ecol. Sociobiol.* 59, 12-23. 10.1007/s00265-005-0003-1

[JEB231829C37] Laska, M. (1990a). Olfactory discrimination ability in short-tailed fruit bat, *Carollia perspicillata* (Chiroptera: Phyllostomatidae). *J. Chem. Ecol.* 16, 3291-3299. 10.1007/BF00982099 24263430

[JEB231829C38] Laska, M. (1990b). Olfactory sensitivity to food odor components in the short-tailed fruit bat, *Carollia perspicillata* (Phyllostomatidae, Chiroptera). *J. Comp. Physiol. A* 166, 395-399. 10.1007/BF00204812

[JEB231829C39] Leiser-Miller, L. B., Kaliszewska, Z. A., Lauterbur, M. E., Mann, B., Riffell, J. A. and Santana, S. E. (2020). A fruitful endeavor: scent cues and echolocation behavior used by *Carollia castanea* to find fruit. *Integr. Org. Biol.* 2, obaa007 10.1093/iob/obaa007 PMC7671165 33791551

[JEB231829C40] Liu, A., Papale, A. E., Hengenius, J., Patel, K., Ermentrout, B. and Urban, N. N. (2020). Mouse navigation strategies for odor source localization. *Front. Neurosci.* 14, 218 10.3389/fnins.2020.00218 32265632 PMC7101161

[JEB231829C41] Lomáscolo, S. B., Levey, D. J., Kimball, R. T., Bolker, B. M. and Alborn, H. T. (2010). Dispersers shape fruit diversity in *Ficus* (Moraceae). *Proc. Natl. Acad. Sci. USA* 107, 14668-14672. 10.1073/pnas.1008773107 20679219 PMC2930445

[JEB231829C42] Louis, M., Huber, T., Benton, R., Sakmar, T. P. and Vosshall, L. B. (2008). Bilateral olfactory sensory input enhances chemotaxis behavior. *Nat. Neurosci.* 11, 187-199. 10.1038/nn2031 18157126

[JEB231829C43] Mathewson, R. F. and Hodgson, E. S. (1972). Klinotaxis and rheotaxis in orientation of sharks toward chemical stimuli. *Comp. Biochem.* 42, 79-84. 10.1016/0300-9629(72)90369-6 4402725

[JEB231829C44] Maynard, L. D., Ananda, A., Sides, M. F., Burk, H. and Whitehead, S. R. (2019). Dietary resource overlap among three species of frugivorous bat in Costa Rica. *J. Trop. Ecol.* 35, 165-172. 10.1017/S0266467419000129

[JEB231829C45] Moore, P. A., Scholz, N. and Atema, J. (1991). Chemical orientation of lobsters, *Homarus americanus*, in turbulent odor plumes. *J. Chem. Ecol.* 17, 1293-1307. 10.1007/BF00983763 24257791

[JEB231829C46] Murlis, J., Willis, M. A. and Cardé, R. T. (2000). Spatial and temporal structures of pheromone plumes in fields and forests. *Physiol. Entomol.* 25, 211-222. 10.1046/j.1365-3032.2000.00176.x

[JEB231829C47] Parolin, L. C., Mikich, S. B. and Bianconi, G. V. (2015). Olfaction in the fruit-eating bats *Artibeus lituratus* and *Carollia perspicillata*: An experimental analysis. *An. Acad. Bras. Cienc.* 87, 2047-2053. 10.1590/0001-3765201520140519 26536853

[JEB231829C48] Porter, J., Craven, B., Khan, R. M., Chang, S.-J., Kang, I., Judkewitz, B., Volpe, J., Settles, G. and Sobel, N. (2007). Mechanisms of scent-tracking in humans. *Nat. Neurosci.* 10, 27-29. 10.1038/nn1819 17173046

[JEB231829C49] Rieger, J. F. and Jakob, E. M. (1988). The use of olfaction in food location by frugivorous bats. *Biotropica* 20, 161-164. 10.2307/2388189

[JEB231829C50] Surlykke, A., Ghose, K. and Moss, C. F. (2009). Acoustic scanning of natural scenes by echolocation in the big brown bat, *Eptesicus fuscus*. *J. Exp. Biol.* 212, 1011-1020. 10.1242/jeb.024620 19282498 PMC2726860

[JEB231829C51] Svensson, G. P., Strandh, M. and Löfstedt, C. (2014). Movements in the olfactory landscape. In *Animal Movement Across Scales* (ed. L.-A. Hansson and S. Akesson), pp. 45-66. Oxford University Press.

[JEB231829C52] Takasaki, T., Namiki, S. and Kanzaki, R. (2012). Use of bilateral information to determine the walking direction during orientation to a pheromone source in the silkmoth *Bombyx mori*. *J. Comp. Physiol. A* 198, 295-307. 10.1007/s00359-011-0708-8 22227850

[JEB231829C53] Tang, Z. H., Sheng, L. X., Parsons, S., Cao, M., Liang, B. and Zhang, S. Y. (2007). Fruit-feeding behaviour and use of olfactory cues by the fruit bat *Rousettus leschenaulti*: an experimental study. *Acta Theriol. (Warsz).* 52, 285-290. 10.1007/BF03194224

[JEB231829C54] Thesen, A., Steen, J. B. and Doving, K. B. (1993). Behaviour of dogs during olfactory tracking. *J. Exp. Biol.* 180, 247-251. 8371085 10.1242/jeb.180.1.247

[JEB231829C55] Thiele, J. and Winter, Y. (2005). Hierarchical strategy for relocating food targets in flower bats: spatial memory versus cue-directed search. *Anim. Behav.* 69, 315-327. 10.1016/j.anbehav.2004.05.012

[JEB231829C56] Thies, W., Kalko, E. K. V. and Schnitzler, H. U. (1998). The roles of echolocation and olfaction in two Neotropical fruit-eating bats, *Carollia perspicillata* and *C. castanea*, feeding on Piper. *Behav. Ecol. Sociobiol.* 42, 397-409. 10.1007/s002650050454

[JEB231829C57] Toelch, U., Stich, K. P., Gass, C. L. and Winter, Y. (2008). Effect of local spatial cues in small-scale orientation of flower bats. *Anim. Behav.* 75, 913-920. 10.1016/j.anbehav.2007.07.011

[JEB231829C58] Vergassola, M., Villermaux, E. and Shraiman, B. I. (2007). “Infotaxis” as a strategy for searching without gradients. *Nature* 445, 406-409. 10.1038/nature05464 17251974

[JEB231829C59] Vickers, N. J. (2000). Mechanisms of animal navigation in odor plumes. *Biol. Bull.* 198, 203-212. 10.2307/1542524 10786941

[JEB231829C60] Von Helversen, O., Winkler, L. and Bestmann, H. J. (2000). Sulphur-containing “perfumes” attract flower-visiting bats. *J. Comp. Physiol. A.* 186, 143-153. 10.1007/s003590050014 10707312

[JEB231829C61] Wachowiak, M. (2011). All in a sniff: Olfaction as a model for active sensing. *Neuron* 71, 962-973. 10.1016/j.neuron.2011.08.030 21943596 PMC3237116

[JEB231829C62] Weissburg, M. J. and Zimmer-Faust, R. K. (1994). Odor plumes and how blue crabs use them in finding prey. *J. Exp. Biol.* 197, 349-375. 7852909 10.1242/jeb.197.1.349

[JEB231829C63] Winter, Y. and Stich, K. P. (2005). Foraging in a complex naturalistic environment: capacity of spatial working memory in flower bats. *J. Exp. Biol.* 208, 539-548. 10.1242/jeb.01416 15671342

[JEB231829C64] Yoh, N., Syme, P., Rocha, R., Meyer, C. F. J. and López-Baucells, A. (2020). Echolocation of Central Amazonian ‘whispering’ phyllostomid bats: call design and interspecific variation. *Mammal Res.* 65, 583-597. 10.1007/s13364-020-00503-0

