# Supplementary material for: Olfactory tracking strategies in a neotropical fruit bat
Source: J Exp Biol. 2021 Feb 19;224(4):jeb231829. doi: 10.1242/jeb.231829 (PMC7904095; doi:10.1242/jeb.231829)
Supplement: Supplementary information [file jexbio-224-231829-s1.pdf]

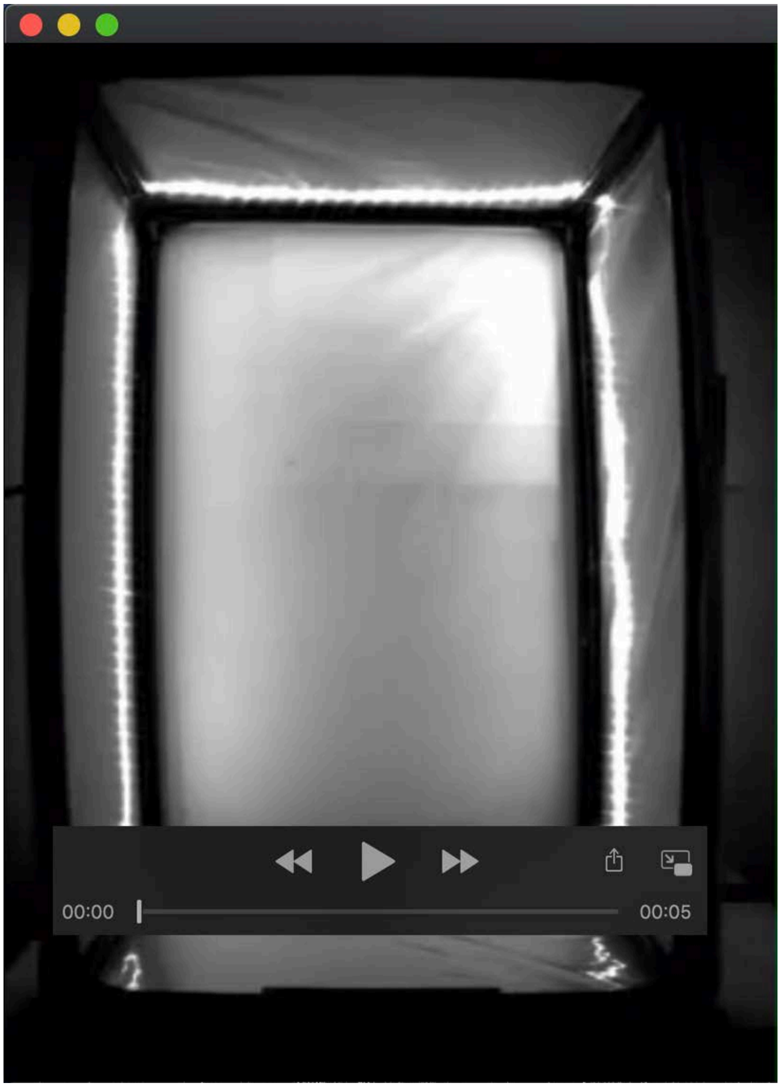

Movie 1. Search behavior of crawling *Sturnira parvidens* locating an odor reward. Real time video of a bat navigating the olfactory assay, following initial placement into the arena. Tracking did not begin until after the cage was closed. The search path tracing was generated automatically using EthoVision XT 13. Color represents instantaneous velocity, with dark blue representing slower time points. Trials were stopped when the bat touch the dish or reward with its nose or mouth. Video was filmed at 30 frames per second.

Journal of Experimental Biology • Supplementary information

Table S1. A Microsoft Excel file containing all data included the presented analyses, including raw data from all choice trials, the subset of trials used for trajectory analysis, raw observations of head scanning behavior, and the original, rotated, and transformed coordinates used in Figure 4C (plots of the 25 – 35 cm decision distance trials).

Click here to Download Table S1
